# Functional Electrical Stimulation Controlled by Motor Imagery Brain-Computer Interface for Rehabilitation

**DOI:** 10.3390/brainsci10080512

**Published:** 2020-08-02

**Authors:** Inchul Choi, Gyu Hyun Kwon, Sangwon Lee, Chang S. Nam

**Affiliations:** 1 Fitts Department of Industrial and Systems Engineering, North Carolina State University, Raleigh, NC 27695, USA; ichoi3@ncsu.edu; 2 Graduate School of Technology & Innovation Management, Hanyang University, Seoul 04763, Korea; ghkwon@hanyang.ac.kr; 3 Department of Interaction Science, Sungkyunkwan University, Seoul 03063, Korea; upcircle@skku.edu

**Keywords:** brain–computer interface (BCI), functional electrical stimulation (FES), sensorimotor rhythm (SMR), adaptive learning, rehabilitation

## Abstract

Sensorimotor rhythm (SMR)-based brain–computer interface (BCI) controlled Functional Electrical Stimulation (FES) has gained importance in recent years for the rehabilitation of motor deficits. However, there still remain many research questions to be addressed, such as unstructured Motor Imagery (MI) training procedures; a lack of methods to classify different MI tasks in a single hand, such as grasping and opening; and difficulty in decoding voluntary MI-evoked SMRs compared to FES-driven passive-movement-evoked SMRs. To address these issues, a study that is composed of two phases was conducted to develop and validate an SMR-based BCI-FES system with 2-class MI tasks in a single hand (Phase 1), and investigate the feasibility of the system with stroke and traumatic brain injury (TBI) patients (Phase 2). The results of Phase 1 showed that the accuracy of classifying 2-class MIs (approximately 71.25%) was significantly higher than the true chance level, while that of distinguishing voluntary and passive SMRs was not. In Phase 2, where the patients performed goal-oriented tasks in a semi-asynchronous mode, the effects of the FES existence type and adaptive learning on task performance were evaluated. The results showed that adaptive learning significantly increased the accuracy, and the accuracy after applying adaptive learning under the No-FES condition (61.9%) was significantly higher than the true chance level. The outcomes of the present research would provide insight into SMR-based BCI-controlled FES systems that can connect those with motor disabilities (e.g., stroke and TBI patients) to other people by greatly improving their quality of life. Recommendations for future work with a larger sample size and kinesthetic MI were also presented.

## 1. Introduction

Healthy individuals whose brains and neuromuscular systems enable normal motor functions can naturally perform Activities of Daily Living (ADLs). Nonetheless, for some people who have disabilities in these functions due to injury or disease, simple tasks become very difficult or impossible to do. To assist this population, researchers in many fields, from physical therapy to engineering, have developed various rehabilitation technologies that help them perform ADLs [1,2]. One such technology, Functional Electrical Stimulation (FES), delivers electrical impulses to either paralyzed or impaired limbs to generate artificial muscle contraction [3,4]. In this way, FES helps disabled people perform ADLs such as walking, reaching, and grasping [5,6]. Some FES devices are controlled by brain–computer interfaces (BCIs), sometimes called brain–machine interfaces.

In general, BCIs can help people communicate and control devices and applications without using peripheral nerves and muscle pathways [7]. BCIs are also a potential method to promote the independence of physically disabled people by means of the BCI’s ability to bypass non-functional neural pathways [8]. A sensorimotor rhythm (SMR)-based BCI-controlled FES system is a novel technology that combines the advantages of FES and BCI systems, and allows severely disabled patients to restore motor functions through the FES system by translating voluntary Motor Imagery (MI) to physical action [9]. There are many potential benefits of combining SMR-based BCIs and FES systems, such as the promotion of neuroplasticity [10], the restoration of motor functions by using voluntary motor intentions [9,11], and providing proprioceptive sensory feedback as a result of their intentions [12].

Although SMR-based BCI-controlled FES methods seem promising, current studies still have central issues: (1) ambiguous instruction of MI tasks during training under SMR-based BCI systems, and (2) difficulties in classifying voluntary MI-evoked SMRs and FES-driven passive-movement-evoked SMRs when FES is activated. Moreover, (3) only a few studies have examined the feasibility of classifying two different MI tasks of a single hand, such as grasping and opening, and (4) few studies have examined human factors and ergonomics (HF/E) perspectives such as subjective mental workload and user satisfaction in the use of SMR-based BCI-controlled FES systems. This research that is composed of two phases was conducted to address these issues by developing a new SMR-based BCI system with visual guidance during training to classify a 2-class MI task in a single hand, as well as voluntary and passive SMRs (Phase 1), and evaluating the feasibility of the proposed BCI-controlled FES system by performing sequential goal-oriented tasks with stroke and TBI patients (Phase 2).

The remainder of this article consists of five more sections (this introduction being Section 1): Section 2 describes a survey of current SMR-based BCI studies for FES systems to identify the limitations of current research and clarifies the current state of BCI-controlled FES technologies. Section 3 presents Phase 1, where an SMR-based BCI system to control FES was developed and validated to address the issues on current research studies. Section 4 describes Phase 2, which assessed the feasibility of the proposed BCI-FES system by conducting a sequential task with fixed order under a semi-asynchronous mode. Section 5 discusses the findings of the present research along with implications and future directions.

## 2. Background

### 2.1. FES Rehabilitation for Stroke and TBI Patients

Each year, more than 795,000 stroke patients suffer a new or recurrent stroke in the United States, and 33 million patients suffer strokes worldwide [13]. In addition, 235,000 severe traumatic brain injuries (TBI) occur in the United States each year, and there are 57 million TBIs worldwide [14]. Many of these patients are susceptible to a combination of significant motor, sensory, and cognitive deficits [15], and they experience residual functional impairments [16]. For instance, 25% to 62% of stroke survivors and 77% of severe TBI patients suffer from major physical complications such as spasticity and muscle weakness [17]. These neuromuscular disorders cause upper and/or lower extremity impairments, such as hemiparesis or hemiplegia, and they hinder patients from performing ADLs naturally. For example, between 25% and 74% of the stroke survivors in the United States (roughly 6.6 million) and worldwide (over 50 million) remain partially or fully dependent on caregivers for ADLs [16]. For this reason, many research studies and intervention methods for these patients have been attempted to aid motor rehabilitation, including physical, neurosurgical, and pharmacological treatments [18].

The physical treatments include repeated range-of-motion exercises [19], thermotherapy [20], and electrical stimulation [21,22]. Among these physical treatment methods for patients, FES is a common adjuvant therapy that has been widely adopted as a clinical application [16,23]. Repeated electrical stimulation could reduce spasticity and improve motor functions in hemiparetic patients [24]. Conventional rehabilitation (i.e., occupational therapy or OT to help patients with a disability be as independent as possible in all areas of their lives) with FES treatment showed better rehabilitation outcomes than OT alone, with respect to reducing spasticity and improving muscle strength and motor recovery in stroke patients [25]. FES systems also have unique strengths in restoring motor functions for hemiparetic patients [26]. First, FES systems have advantages over customized orthoses and exoskeletons because they are lightweight and affordable, and they are universal in terms of the body shape and size of body parts they can accommodate [27]. Other advantages of FES treatment include the promotion of motor learning and neural reorganization [4,28]. The importance of the FES treatment is that cortical activation could be facilitated by forcing the patients to practice with impaired extremities, which patients do not tend to use due to difficulties [29]. Moreover, the psychological benefits of utilizing FES rehabilitation, such as increasing self-esteem and reducing depression [30]. Therefore, studies have been conducted with FES for the motor function restoration of stroke and TBI patients [21,31].

The FES methods can be applied to various body parts, such as the foot [25], shoulder and elbow [32], and forearm [33]. Among them, the restoration of hand function is one of the most important things for patients’ independence in performing ADLs such as feeding, dressing, bathing, and making transfers [34]. Further support for the importance of the hand function were found in [18]. The authors quantified the number of studies of each intervention target and found 115 studies concerning hand and arm functions, as well as 9, 14, and 68 studies for sit-to-stand, standing balance, and gait rehabilitation for lower limbs, respectively.

The FES system can be activated by either push button control, cyclic programs, or the patient’s effort [10,22]. However, when FES is controlled by either automated cyclic programs or physical therapists, the patient’s intention and effort is decreased. This means that the restoration of motor functions is not directly involved in the central nervous system, but it is passively initiated by other factors rather than a patient’s effort. In this case, the neuroplasticity of the patient, which is important to benefit rehabilitation, may not be promoted well due to the lack of synchronization between a patient’s effort and physiological feedback. Neuroplasticity occurs throughout the central nervous system [8], and it is defined as the ability of the human brain to alter its structure in response to environmental demands [35]. Many research studies show the evidence of a positive effect on rehabilitation outcomes when the patient’s intentions are synchronized with the physiological feedback. [36] showed that receiving OT and FES treatments with electromyographic (EMG) biofeedback improved upper extremity function significantly more than receiving only OT and FES.

### 2.2. SMR-Based BCI-Controlled FES Systems

BCI is “a communication system in which messages or commands that an individual sends to the external world do not pass through the brain’s normal output pathways of peripheral nerves and muscles” ([7], p. 769). BCIs are a potential method to promote the independence of disabled persons because of the BCI’s ability to bypass non-functional neural pathways [8]. In particular, to support patients’ mobility and accessibility, a diverse set of BCI applications has been developed, such as BCI-controlled wheelchairs, orthoses, prostheses, and exoskeletons [37,38,39,40,41,42,43,44,45] using various brain imaging technologies (e.g., electroencephalography (EEG), magnetoencephalographic (MEG), functional near-infrared spectroscopy (fNIRS), functional magnetic resonance imaging (fMRI), and positron emission tomography (PET)) [46,47]. Among the various brain imaging methods, the EEG method has been most well-studied because of its advantages such as low prices, convenience, mobility, large cortical coverage, and high temporal resolution compared to other methods [48].

One of the most studied BCI systems is SMR-based BCIs, which utilize MI, an imagined rehearsal of a motor act without any overt movement [49]. SMRs induced by MIs are characterized by (de)synchronization in the alpha and beta frequency bands over the bilateral, contralateral, and ipsilateral motor cortex areas [50,51]. In 1977, Pfurtscheller first introduced the terminology Event-Related Desynchronization (ERD) to describe event-related attenuation in the EEG signal [52]. He then added the term Event-Related Synchronization (ERS) to explain event-related enhancement [53]. These SMR features, ERD and ERS, have been widely employed to decode different MIs, such as left or right-hand motor intention [54]. SMR-based BCIs allow users who have severe motor disability (e.g., the paralyzed) to control physical or virtual devices by decoding their motor intention or motor imagery (e.g., squeezing the left hand or the right hand to move a mouse cursor on the computer screen to the left or the right, respectively) using SMRs induced by the motor imaginations [9].

Combining SMR-based BCIs and FES systems to help severely or completely paralyzed patients is a new approach. This combination could not only restore motor functions [9], but it also has the potential to facilitate neuroplasticity by performing MIs [10]. Furthermore, BCI-controlled FES systems can provide the result of the imagined motion through proprioceptive sensory feedback [12]. For instance, [55] utilized the brain signals to trigger FES during index finger movement training, and the participant showed the recovery of the volitional isolated index finger movements. In addition, [56] applied an SMR-based BCI-controlled FES system to perform dorsiflexion of the paralyzed ankle in a patient with stroke. The authors reported a promising result in that both the amplitude of EMG in the affected tibialis anterior muscle and the range of movement at the ankle joint significantly increased with the SMR-based BCI-controlled FES system in comparison with FES alone. [10] compared five different interventions, such as (1) BCI-controlled FES, (2) EMG-controlled FES, (3) conventional push button-controlled FES, (4) voluntary grasping, and (5) BCI-guided voluntary grasping with 10 healthy participants to investigate the effects on neuroplasticity. More support for the use of SMRs induced by MIs as promising interventions to improve motor function rehabilitation in stroke patients was found in [57]. There were also many studies showing positive outcomes in using SMR-based BCIs for the rehabilitation of patients with severe motor impairments [8,58].

### 2.3. Limitations of Current SMR-Based BCIs for FES

Although SMR-based BCI-controlled FES systems seem promising for the rehabilitation of patients with stroke and TBI, there are still three main limitations in recent research studies. Firstly, most of the current research studies have not clearly described the procedures of MI training in the BCI systems. Brain signals vary not only from person to person, but within the same person due to the non-stationarity of brain signals [59,60]. Thus, machine learning techniques with a set of training procedures have been adopted to improve BCI performance [61]. Although there are many studies that have strived to reduce the training period [62], SMR-based BCI systems still require relatively longer training than other BCI technologies, such as steady-state evoked potential or Event-Related Potential (ERP). In addition, since MI tasks to evoke SMR are mental imaginations that do not involve physical feedback, experimenters or physical therapists cannot know whether the patient is properly performing the MI tasks. To address these issues, users should be provided with clear MI procedures for easy and efficient training. However, only a few research studies focus on these issues [63,64].

Secondly, SMR-based BCIs using EEG signals currently have limited ability to classify two different MIs in a single hand, such as grasping and opening, due to the low accuracy of the current classification algorithms [9]. Some SMR-based BCI studies have classified two different motor functions in a single hand by applying contralateral MIs, such as a right-hand MI for grasping and a left-foot MI for extension. However, this approach may be not able to facilitate neuroplasticity completely due to unnatural control. It is also difficult to distinguish between voluntary MI-evoked SMRs and FES-driven passive-movement-evoked SMRs, because both conditions elicit similar brain activity [50]. This result implies that it is difficult to stop or keep electrical stimulation by using SMR features because brain signals contain voluntary MI-evoked SMRs or passive motion-evoked SMRs mixed with strong electrical artifacts.

Lastly, due to the lack of functionality of off-the-shelf FES units, it is difficult to produce natural motor functions. Many commercially available FES systems have only two or four electrodes to deliver electrical stimulation, so the application of electrical stimulation to multiple muscles and nerves for natural hand and wrist movements is limited. Beyond this, only a few FES systems support real-time computer control that is essential for synchronizing MI-evoked brain signals and FES-driven physical feedback. Although some medical and research purpose-built FES systems support multi-channel electrodes and real-time control, these systems are either expensive or not commercially available in the United State.

## 3. Phase 1: Development and Evaluation of an SMR-Based BCI

### 3.1. Objectives and Hypotheses

The objective of Phase 1 was to address four main limitations of current research studies, as identified in the literature review: (1) unstructured SMR training guidelines; (2) the lack of studies that classify a 2-class MI task in a single hand; (3) the lack of studies utilizing voluntary motor intentions to stop FES; and (4) unclear frequency band selection for SMR. Specifically, a synchronous, cue-based BCI experiment was conducted with stroke and TBI patients to address the following questions: (1) Is it feasible to classify a 2-class MI task such as grasping or opening in a single hand? (2) Is it feasible to use SMR features evoked by voluntary MI to stop or keep FES? (3) What effect does the existence of electrical stimulation have on task performance? (4) Will the ensemble algorithms increase the classification accuracy when compared to traditional classification algorithms such as Linear Discriminant Analysis (LDA) and Support Vector Machine (SVM)? The following hypotheses were formulated to answer the research questions:

**Hypotheses 1.1** **(H1.1):** *The classification accuracy to classify a 2-class MI task in a single hand will be significantly higher than the true chance level.*

**Hypotheses 1.2** **(H1.2):** *The classification accuracy to decoding voluntary MI-evoked SMRs and FES-driven passive-movement-evoked SMRs will be significantly higher than the true chance level.*

**Hypotheses 1.3** **(H1.3):** *The classification accuracy of the ensemble method will be significantly higher than that of the LDA and SVM algorithms.*

### 3.2. Methods

#### 3.2.1. Participants

A total of eight stroke patients (three females; mean age or M = 46.0 ± 13.5 years) were recruited from local rehabilitation centers and clinics. All studies were reviewed and approved by the Institutional Review Board of North Carolina State University. The participants received monetary compensation for participation, and any participants who were displeased during the studies could cease at any time, but no one had chosen to withdraw the study until the studies were complete. The inclusion criteria in this study were (1) suffering from upper limb hemiparesis, weakness on one side of the body, the most common impairment after stroke or TBI; (2) in the chronic state of stroke, the condition of a stroke patient persisted without recurrent strokes for more than three months, to prevent the potential risk of recurrent stroke, and (3) having normal sensitivity to feel pain and discomfort on the impaired forearm.

#### 3.2.2. Visually Guided Instructions for MI Tasks

Since MI tasks do not involve physical movements, it is difficult for the experimenter to know whether the participant is performing MI tasks in the correct way. To address this issue, clear instructions are required to help participants during training. Thus, video clips were provided in this study as a visual guideline for MI tasks, and participants were asked to follow the movie clips to train them in becoming familiar with the MI tasks.

The visual guideline consisted of two video clips to represent Slow One-time Grasping (SOG) and Fast Cyclic Opening (FCO). The video clip of SOG displayed a slow one-time grasping for three seconds (1/3 Hz) starting at a neutral position with a straight hand and wrist, while that of FCO showed a three-time repetition of an opening motion (from a grasping position) for three seconds (1 Hz). Then, participants were asked to imagine each motion depicted in the video clip as similar as possible. The two different rhythmic motions used in visual guidance were intended to classify a 2-class MI task in a single hand utilizing distinct SMR features evoked by different rhythmic MIs [65,66]. These video clips were displayed on a 21-inch liquid-crystal display (LCD) monitor located approximately 42 inches in front of participants, and the centerline of the monitor was set within 10 degrees of eye level to follow the guideline of viewing distance and screen size, and viewing angle for individuals, respectively [67].

#### 3.2.3. Procedure

FES electrodes were placed on the adequate coordinates with the user-specific FES parameters identified [68]. Then, the anti-static wrist strap was applied to the opposite hand of the affected forearm with the FES electrode to ground out the electrical stimulation. Afterward, the participants were then asked to wear an EEG cap with 32 active EEG electrodes (g.tec medical engineering GmbH, Graz, Austria). When the FES and BCI systems were ready, participants were given detailed explanations of the synchronous experiment and were instructed on how to perform the MI tasks according to the visual guideline shown on the monitor.

**Figure 1:**
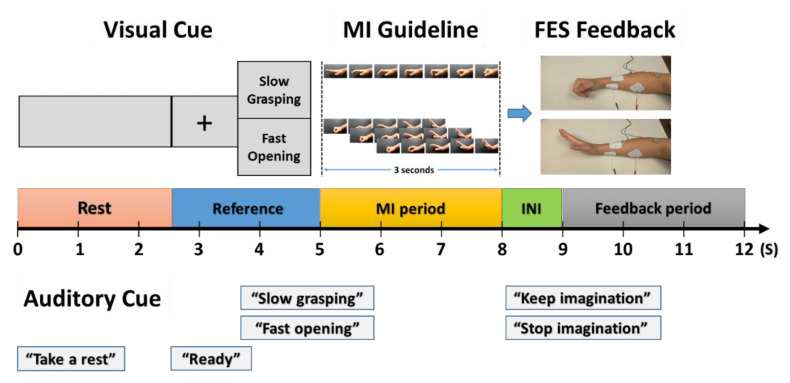
Schematic illustration of the experiment procedure. Text in the blue box indicates the auditory cue that played at the beginning of each period, and INI is an abbreviation of the Functional Electrical Stimulation (FES) initiation period. MI: Motor Imagery.

The experiment in Phase 1 consisted of five sessions with 24 trials per session, and there was a three-minute break between sessions. Each trial lasted 12 s and consisted of a rest period (2.5 s), a reference period with cues (2.5 s), an MI period (3 s), an FES initiation period (1 s), and a feedback or FES period (3 s), as shown in Figure 1. During the rest period, the screen showed only the gray background without any image, and an auditory cue (“take a rest”) was played at the beginning of the rest period. Participants were required to remain calm during this period to stabilize EEG signals. Then, the reference period was followed by a cross fixation screen and an auditory cue (“ready”) at the beginning of the period, and the visual (text) cue and the auditory cue for either “slow grasping” or “fast opening” were presented at the end of the reference period. The participants were asked to focus on the screen to be ready for MI tasks. Afterward, the MI period was successively given to participants with the visual guideline of SOG or FCO for three seconds. During this period, the participants were asked to mimic MI tasks as close as possible by following the visual cues. After the MI period, the FES initiation period lasted for 1 s, during which the FES system activated the same motion to the subjects’ affected forearm as presented in the visual guideline. In this period, the current amplitude of FES was gradually increased by utilizing the ramp time function to improve user satisfaction. At the end of the FES initiation period, the feedback period was successively given to the participants. In this period, the participants were required to keep or stop the MI tasks given in the feedback period according to auditory cues such as “keep imagination” or “stop imagination”, respectively.

In the MI period, participants conducted a total of 120 trials including 60 trials for each SOG and FCO, while they performed 60 trials for either keep or stop MI tasks in the feedback period. All trials were pseudo-randomized so that the number of trials for different conditions were balanced with each combination. The total time of the experiment was around 40 min, and the completion time of Phase 1 was 60 min, including the setup time.

#### 3.2.4. Signal Acquisition and Processing

Signal acquisition: EEG signals were measured from 32 active electrodes (e.g., AFz, F7, F3, Fz, F4, F8, FC5, FC3, FC1, FC2, FC4, FC6, T7, C5, C3, Cz, C4, C6, T8, TP7, CP5, CP3, CPz, CP4, CP6, TP8, P3, P4, PO3, PO4, O1, and O2) with the ground at Fpz and the left earlobe reference. EEG signals were sampled at 256 Hz via two g.USBamp biosignal amplifiers (g.tec medical engineering GmbH, Austria), and notch-filtered at 60 Hz to remove electrical mains hum in the United States with the BCI2000 system. For details, see [69]. Then, the recorded EEG signals were divided into three subsets for further analysis as follows: (subset 1) the reference and MI period for ERD/ERS and Common Spatial Pattern (CSP) analyses to detect the existence of MI-induced SMRs when FES was not being applied; (subset 2) the MI period for the CSP method to classify a 2-class MI task in a single hand, and (subset 3) the feedback period for the CSP analysis to decode the existence of SMRs when FES was being applied. The CSP method transforms multi-channel EEG data into a subspace using a variance matrix that can maximize discrimination between two classes (i.e., two motor imageries, C1 and C2) [for details, see 74]. For example, the normalized covariance matrix was calculated from

(1) $$C_{C1}=\frac{X_{C1}X_{C1}^{T}}{trace\left(X_{C1}X_{C1}^{T}\right)},\;C_{C2}=\frac{X_{C2}X_{C2}^{T}}{trace\left(X_{C2}X_{C2}^{T}\right)},\;\;C_{c}=C_{C1}+C_{C2}$$

where trace (X_Cx_X_Cx_^T^) is the sum of diagonal elements of (X_Cx_X_Cx_^T^). Afterward, the composite spatial covariance, C, and projection matrix, W, were calculated from $C_{c}=C_{C1}+C_{C2}=U_{c}\lambda U_{C}^{T}$ W = U^T^$\sqrt{\lambda }U_{C}^{T}$, where U_c_ is a matrix of normalized eigenvectors, and λ is eigenvalues. Then, the projection matrix W transformed EEG signals into two CSP features.

Signal preprocessing: Once the brain signals were recorded, noise, artifacts, and other irrelevant brain signals were filtered out. EEG signals were visually inspected for unexpected EEG contamination usually caused by body movements and electrode drifts during the experiments, and those trials were excluded from further processing. In addition, EEG signals were filtered by an automatic artifact rejection toolbox from the EEGLAB. For details, see [70,71]. The rejection thresholds were set at 150 μV for the large fluctuation threshold and 3 standard deviations for the probability threshold of entropy and kurtosis. The remaining trials were then band-pass filtered between 1 and 29 Hz to remove irrelevant brain signals such as muscle artifacts. Afterward, principal component analysis (PCA) was applied to the filtered EEG signals to project high-dimensional data (32 channels) into lower dimensions while maintaining important brain features. After applying PCA, eigenvectors and eigenvalues of a covariance matrix were generated. Among all eigenvalues, the first few components that could explain 95% of variance were selected with the assumption that the important brain features are contained in those few components. This procedure not only addresses the high dimensionality issue but also helps to speed up an independent component analysis (ICA) decomposition process by reducing the dimension of the EEG signal.

The reduced dimensional EEG signals after the PCA procedure still mixed with irrelevant independent components such as eye-blinking artifacts and visual stimulus-evoked potentials. Thus, an ICA method was applied to retain only the relevant independent components by removing irrelevant components. After the ICA decomposition process, the EEG signals were decomposed into maximally independent components, and the artifact (irrelevant) components could be identified by plotting each independent component in a component activity time course and a topographical map. After removing the artifact-related components, EEG signals were projected back with the whitened data. Finally, the surface Laplacian estimation was applied to the EEG signals between the reference and MI periods (EEG subset 1) to enhance the spatial EEG traces of the ERD/ERS procedure [72].

Feature Extraction: After the signal preprocessing procedure, two feature extraction methods were utilized, including ERD/ERS [73] and CSP methods [74]. The ERD/ERS method was applied only to EEG subset 1, while the CSP was analyzed for all three EEG subsets. To compute ERD/ERS, the preprocessed EEG subset including both SMR and reference periods was band-pass filtered with a width of 4 Hz by FIR filter (For details, see [75]). Then, the EEG signals were divided into seven sub-bands from the first sub-band bin, [1,2,3,4,5] Hz, to the seventh sub-band bin (for details, see [25,26,27,28,29]), Hz, with 4 Hz intervals. Afterward, the EEG data of each sub-band bin were transformed from time-domain data to frequency-domain data by using Fast Fourier Transform (FFT) with the Hamming windowing method for 2.5-s data epoch (640 data points). Afterward, the relative band power of each sub-band bin, ERD/ERS, was computed with the value of FFT in the reference period and that in the MI period for each trial by following [76].

The three EEG subsets were analyzed with the sub-band CSP (SBCSP), which can project a multidimensional EEG dataset into a low-dimensional subspace. SBCSP allows to decompose the EEG subsets into seven sub frequency bands by following the same procedure utilized in the ERD/ERS method. From each sub-band bin, the eigenvalues were calculated and utilized to determine the best feature from the projected EEG matrix. Then, the features from SBCSP were utilized to increase the subsequent classification accuracy by fusing the classification scores of each sub-band feature. The advantages of SBCSP include (1) not requiring an exhaustive band selection procedure and (2) allowing the use of multiple frequency bands at the same time [77].

Classification: Three different classification algorithms, Fisher’s LDA, SVM, and ensemble methods, were tested to identify the best classification algorithm and classifier parameters. Many BCI studies have reported good classification outcomes with LDA, as well as have validated these methods as among the most suitable algorithms for SMR feature classification [78]. For the SVM classification algorithm, the kernel function used quadratic components to cover non-linear characteristics in the brain signal, and the least-squares method was applied to find the separating hyperplane. The ensemble method by combining the outputs of the LDA and SVM classifiers was also evaluated to test hypothesis 1.3, and the final decision of the ensemble method was made with the weighted majority voting by combining the decisions of LDA and SVM, as well as by combining the features of each classifier [79]. Each EEG subset was uniformly divided into 10 sub-datasets to apply a 10-fold cross-validation method [80]. Nine sub-datasets were used as training datasets to build a classification weight vector, and the remaining one sub-dataset was utilized as a test dataset to calculate the classification accuracy.

#### 3.2.5. Independent and Dependent Variables

Two independent variables (IVs) were manipulated including a period type and a classification type. The period type had three levels, including SMR, ACT, and FES periods, and each period had different roles as follows; (1) SMR period: detecting the existence of an MI-induced SMRs when FES was not being applied; (2) ACT period: classifying a 2-class MI task in a single hand, and (3) FES period: decoding the existence of SMRs to control FES when FES was being applied. The classification type had three levels for all periods such as LDA, SVM, and ensemble methods.

Accuracy (%) was measured as a dependent variable (DV), which is the ratio of the correct classifications over the number of trials [45]. The results from 10-fold cross-validation for each period were averaged to calculate the accuracy with three classification types [80]. Since the experiment in Phase 1 was a balanced 3 × 3 within-subjects factorial design with two IVs (period type and classification type) with participant as a blocking variable and one DV (accuracy), a univariate ANOVA was used. Prior to conducting two-way ANOVAs on the accuracy data, the assumptions of both homoscedasticity and normality of residuals were assessed in JMP^®^ (SAS institute Inc., Cary, NC, USA). The results of the Shapiro–Wilk test for normality (W = 0. 982593, *p* = 0.4207) and Brown–Forsythe test for homoscedasticity (F_8, 54_ = 0.7150, *p* = 0.6773) showed that the data did not violate the ANOVA assumptions.

### 3.3. Results: Accuracy (%)

Table 1 shows the accuracy of each period and classification method. The period consists of the SMR, ACT, and FES period, while the classification method includes LDA, SVM, and the ensemble method. The accuracy was calculated by averaging the 10-fold cross-validation results as defined before.

The results showed that the average accuracies between three periods were significantly different (*F*_2, 56_ = 47.8111; *p* < 0.00001; ηp^2^ = 0.63066), but the main effect of classifier types and interaction were not significant. Tukey’s honest significance test (HSD) was tested to determine significant differences in the main effects of the MI period. The results showed that the average accuracy of the SMR period was significantly higher than that of the ACT period, and that of the ACT period was significantly higher than that of the FES period. Furthermore, the results of the t-test showed that the average accuracy of the ACT period (68.82%; t23 = 4.10, *p* < 0.001) was significantly higher than the true chance level (57.43%), but the FES period (59.16%; *t*_23_ = 1.17, *p* = 0.2558) was not significant. Tukey’s HSD was also tested to determine significant differences in the main effects of classifier type. Although the average accuracy of the ensemble method was higher than others, the results of the statistical analysis showed that the average accuracies between classifiers were not significantly different.

### 3.4. Discussion

As expected, the results of statistical analysis showed that the classification accuracy of a 2-class MI task was significantly higher than the true chance level. Therefore, the hypothesis H1.1 was rejected in favor of the alternative hypothesis, which states that the classification accuracy to classify a 2-class MI task in a single hand is significantly higher than the true chance level. This result could be extended to any SMR-based BCI system, as well as other SMR-based FES-BCI systems. The results of statistical analysis also showed that the accuracy of the FES period was not significantly higher than the true chance level. Therefore, the hypothesis H1.2 was not rejected. The reasons for these results are as follows. First, the duration of the FES period might be too short to stabilize the excited brain signal due to electrical stimulation. Second, electrical artifacts from the FES might be not completely grounded by the anti-static wrist strap applied to the opposite hand of the affected forearm with the FES electrode.

The results of the classification type showed that the average accuracy of the ensemble method was higher than other methods, but statistical analysis showed that the difference between classification types was not significant. One possible reason is that the accuracy of classification among subjects showed a large variation due to the difference in individual ability. To address this issue, the accuracy of classification was standardized within participants, and ANOVA analysis was conducted again. The results of ANOVA showed that the standardized accuracy between classification types was significantly different (*F*_2, 56_ = 3.347; *p* = 0.0424; ηp^2^ = 0.10677). Therefore, the modified hypothesis of H1.3 was rejected, which means that the standardized classification accuracy of the ensemble method is significantly higher than that of LDA. Finally, two classification features including the user-specific classifier parameter set and a probability weight for the ensemble method were constructed.

## 4. Phase 2: Feasibility of the Proposed BCI-FES System

### 4.1. Objectives and Hypotheses

The objective of Phase 2 was to evaluate the feasibility of the proposed BCI-controlled FES system by addressing the following questions. (1) Is it feasible to use the proposed SMR-based BCI-controlled FES system in an online experiment? (2) Does the existence of electrical stimulation affect task performance? (3) Does the application of adaptive learning affect task performance? More specifically, the following hypotheses were formulated to answer the research questions.

**Hypotheses 2.1** **(H2.1):** *The classification accuracy will be significantly higher than the true chance level.*

**Hypotheses 2.2** **(H2.2):** *Task performance between two periods, the No-FES period and the Yes-FES period, will be significantly different.*

**Hypotheses 2.3** **(H2.3):** *Task performance after applying adaptive learning will be significantly greater than before.*

### 4.2. Methods

#### 4.2.1. Participants

The same patients who participated in Phase 1 continued this experiment either after a 20-min break or when the participants felt ready. Any patients who were displeased with the experiment could cease at any time, but no one had chosen to withdraw.

#### 4.2.2. Experimental Task and Modes

To mimic ADLs, participants were asked to hold, move, and release a small ball from an initial position to a target position by utilizing the proposed SMR-based BCI-controlled FES system. The initial and target positions of the ball were set within the comfortable distance in front of participants. The distance between the initial and the target position was set to 10 cm. There were two different modes of the experiment that are mainly applied in BCI studies [81]. The first mode is a synchronous, cue-based experiment that is the same type of experiment as that performed in Phase 1. In this type of experiment, the participants are usually provided with visual, auditory, or tactile cues and perform tasks accordingly in a fixed time interval. Afterward, through the offline analysis, the measured brain signals are divided into groups according to the cues, and the brain features representing each group are extracted. Then, classifier parameters are generated to best distinguish each group based on the features. The second mode is an asynchronous, self-paced experiment where the users perform a task at their own pace rather than following the cues. The advantage of the asynchronous experiment is that the BCI system can be more realistic and flexible, because it allows the user to perform the desired operation at his/her own pace [82]. It would be very useful for patients to be able to control the impaired body solely through MIs. However, since it is difficult to know when the user performed MI tasks, the asynchronous mode has a critical issue such that the classification accuracy is low due to the high false positive ratio [83]. Due to the low accuracy of the MI tasks because of the high false positive ratio, it is somewhat difficult to perform experiments on patients in a completely asynchronous situation. As a result, asynchronous BCI research has not been studied as much compared to synchronous BCI research [60].

More training in synchronous experiments could help to solve this issue, but long-lasting BCI training is not easy for participants with impaired physical conditions (and/or mental conditions such as attention deficits after stroke and TBI), since it requires participants to sit down and focus on repetitive mental tasks while minimizing movement. Furthermore, the results of synchronous experiments are not always guaranteed to be the same results as those of asynchronous experiments. This is due to cue differences (Yes versus No), analysis time differences (fixed versus variable), and the nature of brain signals that have non-stationarity and inter- and intra-variability [84]. Alternatively, we can consider increasing the accuracy through more practice in the same environment as the asynchronous mode, but there are also the following issues. First, it is difficult to know when the patient has successfully performed MIs, as knowing this information is essential to categorize the EEG signals into the two groups (Yes-SMR versus No-SMR) to initiate the FES system. Second, if the errors, either false positive or false negative, are accumulated, the participants will be easily confused and frustrated. Finally, an additional time required to correct the erroneous movements will increase the duration of the experiment, and we should take into consideration that prolonged FES may increase muscle fatigue in patients.

Thus, Phase 2 used a semi-asynchronous mode for performing a fixed sequence. This mode has three important features that combine the advantages of both synchronous and asynchronous modes as follows: (1) a fixed sequence of tasks to clarify what the user needed to do, (2) error-free results to prevent participants from being confused or exhausted, and (3) initiating the FES system by detecting SMRs similar to the asynchronous mode. Under the proposed semi-asynchronous mode, participants would be free from confusion and frustration while using the SMR-based BCI-controlled FES system at their own pace. Furthermore, the experimenter can enhance the classification performance by adjusting the classifier parameters using the most recently measured brain signals under this mode, which is also known as adaptive machine learning [85,86].

#### 4.2.3. Procedure

**Figure 2:**
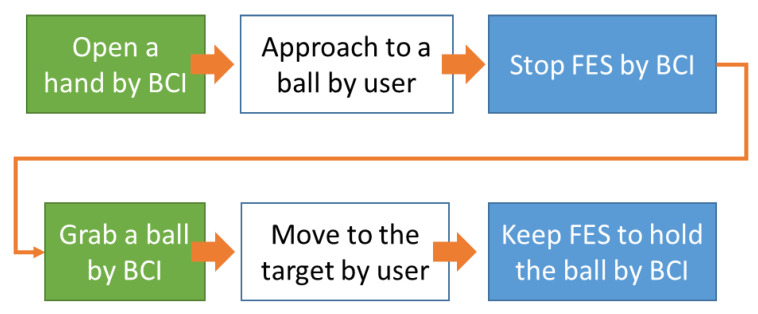
Sequence of the predefined tasks in Phase 2. BCI: Brain-Computer Interface.

Participants were asked to conduct the fixed sequence MI tasks according to a specific goal. The BCI tasks consisted of (1) opening the hand with FCO MIs similar to the previous study; (2) stopping the FES system by not imagining any action similar to “stop imagination”; (3) grasping a ball with SOG MIs; (4) moving the ball to the target position without going through the BCI system, and (5) holding the ball on the target position by continuously imagining SOG. In this experiment, patients were asked to perform all tasks as quickly as possible, except the ‘holding the ball’ task, which should last as long as possible, using the appropriate MIs trained in the previous experiment (see Figure 2).

In Figure 2, the colored boxes represent the tasks that the participant should perform through the BCI system, and the white boxes represent the tasks that the participant was asked to perform without using the BCI system. The green boxes on the left indicate MI tasks without FES, and the blue boxes in the middle show the tasks performed under FES. The following describes each step that participants were asked to perform in detail.

Step 1—Open a hand: First, participants were asked to perform the opening MI task by imagining FCO trained in the previous experiment. Once the BCI system detected an FCO-related SMR, the FES system was activated for an opening motion of 5 s. If the system did not detect the FCO-related SMR in the maximum analysis time (6 s), then the system was automatically started so that the experiment time did not increase excessively. Afterward, the participants were asked to move their hand near a ball to be ready to grab.

Step 2—Stop FES: After five seconds, the participants were asked to stop the FES system as soon as possible without imagining any motion similar to “stop imagination” in Phase 2. When the BCI system detected the absence of MI, the FES system stopped working. To prevent excessive muscle fatigue and discomfort, if participants could not stop the FES system in the maximum analysis time, the system automatically stopped operating. Then, participants were given a 10-s break to stabilize excited brain activity from the physical movements and electrical stimulation, and the BCI system also stopped classifying brain signals during the break.

Step 3—Grab a ball: After the break, the participants were asked to conduct the SOG MIs to grab the ball. If an SOG-related SMR was detected, the FES system was activated for grasping motion for 5 s, and they were asked to move the ball to the target position within 5 s. Similar to Step 1, if the system did not detect the SOG-related SMR in the maximum analysis time, then the system automatically initiated a grasping motion.

Step 4—Hold the ball: After the participants had moved the ball over to the target position, they tried to keep holding the ball for another 5 s. To hold the ball, the grasping SMR should be maintained when FES was activated for grasping. If the grasping SMR was successfully maintained during the maximum analysis time, the FES system automatically stopped for safety. This was the task sequence of this experiment necessary to complete one trial.

Participants were asked to conduct two sets of the sequential tasks in each trial, which lasted until all tasks were finished. A total of 20 trials were conducted during the experiment. The completion time varied due to the different performance levels between participants, but the average completion time was around 40 min.

### 4.3. Signal Acquisition and Processing

Signal Acquisition and Preprocessing: The signal acquisition and preprocessing procedures were identical to Phase 1 except for the artifact removal and ICA procedure, because these procedures require additional computational times and visual inspections that are not suitable for real-time experiments [87].

Feature Extraction and Selection: CSP and ERD/ERS features used a user-specific CSP projection matrix and ERD/ERS weighted matrix obtained as the results of Phase 1.

Classification: For the online classification, the ensemble method was utilized, as the results of the previous study showed that performance of the ensemble method was significantly higher than the LDA method. During the experiment, EEG signals were categorized as three periods similar to Study 1, including the SMR period (to detect the SMR features to initiate the FES system), the ACT period (classifying different MIs including grasping and opening), and the FES period (to either keep or stop the FES system). One difference was that the incoming signal was the same, but the role was changed according to the current goal. For example, if the current status is to try to perform opening a hand, then the current period is defined as the SMR period. If the SMR feature is detected, then the role of the same EEG dataset is changed to the ACT period. Therefore, detecting SMRs and classifying a 2-class MI task are performed sequentially. However, if SMR-related brain features are not detected, the current role is not changed, and the SMR period is maintained. On the contrary, if a target role of either grasping or opening is not detected during the ACT period, then the current status is set back to the SMR period. When the BCI system detects only the target role from the ACT period, the FES system is activated according to the decision from the BCI system.

Adaptive Learning: When participants completed the first 10 trials, the adaptive learning method was applied to adjust classifier parameters. Many different adaptive learning methods are available, and the advantages and computational time of each method vary. More elaborate adaptive learning methods might yield higher accuracy, but they require more computational time. Adaptive learning methods can be divided into two groups, supervised and unsupervised learning. As [85] recommended due to ease and good performance, the Pooled mean (PMean) adaptive estimation method was chosen. Since this method is the unsupervised adaptive learning method, it can also be utilized in the asynchronous mode. The PMean estimation can be determined as follows:

(2) $$\mu_{t}=\left(1-\eta \right)\mu_{t-1}+\eta x_{t},\;\;\;\;\omega O_{t}=-\omega^{T}\left(\mu_{t}\right)$$

where W0_t_ = the bias of the classifier at trial, η = the updating coefficient (0.1), and x_t_ = the new feature obtained at trial t.

### 4.4. Independent and Dependent Variables

Two IVs were manipulated: FES existence type (No-FES versus Yes-FES) and learning type (before learning versus after learning). The FES existence type indicates the existence of electrical stimulation during MI tasks, and it has two levels: No-FES (opening and grasping, steps 1 and 3, respectively) and Yes-FES (stop and keep, steps 2 and 4, respectively). The learning type indicates the application of the adaptive learning method during the experiment, and the learning type also has two levels: before learning and after learning.

DVs such as task performance (completion rate, accuracy), sensitivity, information transfer rate, and subjective assessment were measured. Task performance and subjective assessment were then statistically analyzed with ANOVA tests. Before conducting ANOVA, two parametric assumptions were assessed in JMP^®^ (SAS institute Inc., Cary, NC, USA), similar to Phase 1. ANOVA assumptions were not violated (Shapiro–Wilk test for normality: W = 0.957758, *p* = 0.2383; Brown–Forsythe test for homoscedasticity: F_3, 28_ = 0.0692, *p* = 0.9759). Thus, a two-way ANOVA was conducted to test the main effects of two IVs (FES existence and learning) and their interaction effects on one dependent variable, accuracy.

Task Performance: (1) Completion rate (%)—A completion rate is defined as how quickly grasping, opening, and stopping FES operation have been performed, or how long it has lasted during the keep-FES task [88,89]. For example, the completion rate will be 100% if the predetermined MI tasks to be performed succeed in only one attempt (0 s). However, if the target SMR cannot be performed before the maximum analysis time (6 s), the completion rate would be 0%. On the contrary, in the case of performing the keep-SMR task, the SMR must be maintained for 6 s, the maximum analysis time, to achieve 100% completion rate. (2) Accuracy (%)—The successful task implies that the MI tasks were performed without error. However, in the case of semi-asynchronous and asynchronous experiments rather than time-locked and cue-based experiments, the definition of error is not clear. This is because if the operation does not occur because the SMR-related features are not detected during the SMR period, it is a delay rather than an error requiring additional work to be corrected. In addition, due to the nature of the asynchronous experiment, it is difficult to distinguish whether the delay is intended due to a failure to implement MIs. However, from another point of view, it is difficult to define this as a successful task performance if the delay caused by MI induction failure is long enough to affect BCI performance. Therefore, the accuracy in this experiment was defined as follows. (1) If an incorrect decision was made during the SMR period (i.e., no SMR was detecting), then it belongs to a delay until the maximum analysis time (6 s). (2) If an incorrect decision was made during ACT (i.e., the target action was ‘opening’, but classified as ‘grasping’, and vice versa), then it belongs to an error. (3) If an incorrect decision was made during the FES period (i.e., the action should be ‘keep’, but it was classified as ‘stop’, and vice versa), then it belongs to an error. The structure of the classification algorithms utilized in this phase also agree on this argument, because even if it fails to detect the SMR function, it is not necessary to correct this error, but only a delay of 1s occurs. (3) Information Transfer Rate: Many BCI studies have utilized Information Transfer Rate (ITR) to evaluate the performance of BCI systems, which can convey the amount of information per time (bits/minute) in terms of the accuracy and speed [90].

*Subjective Assessment:* Participants were asked to complete the NASA Task Load Index (NASA-TLX) questionnaire to measure the subjective mental workload, which is the same as in Phase 2 [91]. Then, the results of studies were analyzed to compare changes in the subjective mental workload between the two experiments.

### 4.5. Results

#### 4.5.1. Task Performance

Table 2 summarizes the completion rate of each task. As the goal of ‘keep FES’ was to maintain grasping FES as long as possible by continuing SOG MI tasks, the completion rate of ‘keep FES’ was calculated. The results showed that the average completion rate of each task including ‘grasping’, ‘opening’, ‘keep FES’, and ‘stop FES’ were 55.94%, 53.44%, 62.08%, and 57.71%, respectively. There were some excessive values between subjects. For example, the completion rates of the ‘opening’ action for subject ‘S04′ and ‘S03′ were 93.3% and 29.2%, respectively. Furthermore, within the participants, there were large variations in the completion rate. For example, Subject ‘S04′ showed the highest completion rate (95%) for the ‘stop FES’ task among all the participants, but that for ‘keep FES’ was the lowest completion rate (13.3%). The average completion rate for the ‘keep FES’ task was lower than other values, and the reason is that the ‘keep FES’ task required six successive successes to obtain a 100% completion rate, while other tasks only needed the first-time success.

Table 3 shows the average completion rate for each IV. The results showed that the average completion rate of the No-FES period was higher than that of the Yes-FES period. Furthermore, the average completion rate after applying the adaptive learning method was higher than before.

The accuracy of each task is shown in Table 4. The results showed that the accuracy of the No-FES period was a little higher than that of the Yes-FES period, but the differences were not large. There was some evidence that the classifiers might be biased to a certain direction because Subject S05 tended to say ‘keep FES’, while Subject S04 tended to say ‘stop FES’.

Table 5 shows the accuracy of each IV, and the results showed that the accuracy of each FES condition increased after applying learning. It indicates that the adaptive learning procedure enhanced the identifiability of both classification algorithms. Between two periods, the improvement of the No-FES period was higher than that of the Yes-FES period.

Table 6 shows the ITR of each IV, and the ITR values increased a little bit after applying the adaptive learning method, but the difference was great. The results of ITR also indicated that the performance of the proposed SMR-based BCI system fell into the average ITR ranges (10–25 bit/min) [7].

An ANOVA was also performed to test the main effects of IVs, the existence of FES, and the learning type and interactions between IVs with participant as a blocking variable on the completion rate and ITR, separately. An ANOVA was also performed to test the main effects of IVs, the existence of FES, and the learning type, and interactions between IVs and the participants as a blocking variable on accuracy. There were significant differences in learning type (*F_1, 21_* = 8.2273; *p* = 0.0092; ηp2 = 0.28149), but the main effect of the FES existence type and interaction effect were not significant. In addition, Tukey’s HSD test showed that the average accuracy during the No-FES period after applying adaptive learning was significantly higher than that during the Yes-FES period before applying adaptive learning.

#### 4.5.2. Workload

To analyze the subjective mental workload changes between the two BCI experiments in Phase 1 and 2, the participants were asked to answer the NASA-TLX questionnaire after each experiment [91]. To test the significance of the differences between the experiments, a two-tailed Student’s t-test was performed with the differences (scores of the second experiment—score of the first experiment) of each question. Table 7 shows that mental demand and frustration increased significantly, while performance significantly decreased compared to the first experiment.

### 4.6. Discussion

#### 4.6.1. The Feasibility of the Proposed BCI-FES System

Phase 2 investigated the feasibility of the proposed BCI-FES system under the semi-asynchronous mode not only for classifying a 2-class MI task in a single hand after detecting the existence of SMR but also for detecting the presence of MI-evoked SMRs when FES was activated. The results showed that the classification accuracy of the No-FES period, detecting the existence of SMR and classifying a 2-class MI task in a consecutive manner after applying the adaptive learning method (61.9%; t = 2.71, *p* = 0.0301) was significantly higher than the true chance level (50.15% with 10 trials for both detecting SMR and classifying a 2-class MI task). However, the classification accuracy of the No-FES period before applying the adaptive learning method (49.4%) was not significantly higher than the true chance level (53.77%). In addition, the accuracies of decoding voluntary and passive SMRs for both learning types (45.0% and 55.0%) was not significantly higher than the true chance level. Therefore, the null hypothesis of H2.1 could be rejected only in the No-FES period after applying adaptive learning, and it is in favor of the alternative hypothesis, which states that the classification accuracy of the No-FES period after applying adaptive learning is significantly higher than the true chance level. Although the classification accuracy of the No-FES period after applying adaptive learning was significantly higher than the true chance level, it did not achieve the threshold level (70%) for effective control through a BCI system [92].

Some reasons to explain these results are as follows. First, the visual cues presented in the synchronous experiments in Phase 2 created an experimental environment that was different than the semi-asynchronous experiments in Phase 2. During the experiment in Phase 1, the video clips were provided to help participants mimic MI tasks, while there was no visual guidance in Phase 2. Therefore, the patients might have confused target MI tasks or missed the timing of MI tasks during the semi-asynchronous experiment. The different decision-making procedures could be another reason. For example, the experiment under the synchronous mode in Phase 1 consisted of three classification periods, known as SMR, ACT, and FES periods, and the accuracies of the SMR periods and ACT periods were separately analyzed. In contrast, in Phase 2, SMR and ACT classifiers were combined in a sequential mode under the semi-asynchronous mode. In this condition, the SMR classifier was for a go–no-go decision similar to a switch, and the ACT classifier classified between grasping and opening only if the SMR classifier determined ‘Go’. Therefore, the accuracy of the final decision can be calculated by multiplying two probabilities. Thus, the expected accuracy of the combined classifiers was 52.4% where the accuracies of the SMR and ACT periods in Phase 1 were 73.49% and 71.25%, respectively, which was similar to the actual accuracy of 52.8%. In addition, the difficulty of classifying a two-class MI task can explain low accuracy. For example, the results of the other study [93] using a 2-class MI task similar to this study showed that the classification accuracy of the offline analysis did not exceed the threshold level (70%).

In addition, the reasons for the low accuracy of classifying voluntary and passive SMRs can be explained similar to Phase 1, such that (1) electrical artifacts from the FES might be not completely grounded, (2) the duration of the FES period might be too short, and (3) it was difficult to stay calm without movement-related imagination when FES was activated as a few participants mentioned.

#### 4.6.2. FES Existence Type

The ANOVA results revealed that there were no significant main effects of the FES existence type on task performance, as well as any interaction effect between the FES existence type and learning type. The results between the two studies were different in that the classification accuracy of the FES period with electrical stimulation was significantly lower than the SMR and ACT periods. One reason for the different results is that each experiment was performed in a different classification procedure as explained previously. As the final decision was made by sequentially combining the results from both SMR and ACT periods, the expected accuracy of the combined classifier (52.4%) decreased compared to that of each period. Furthermore, the maximum analysis time (6 s) might be too short to modulate SMRs during the detecting SMR period. Finally, the sample size might be too small to have enough statistical power. Due to these reasons, the ANOVA results might not show any significant main effect of the FES existence type on task performance.

#### 4.6.3. Learning Type

The statistical analysis confirmed that the application of the adaptive learning method had a significant effect on the classification accuracy, and the post-hoc analysis showed that the application of the adaptive learning method significantly increased the accuracy similar to other studies [61]. However, other task performance, such as the completion rate and ITR, did not show any significant main effect of the learning type. The difference in the experiment environment between offline (with visual cues) and online (without visual cues) experiments can explicate this effect. As the offline experiment utilized visual cues with fixed time intervals, the classifiers also affected the visual stimulus-evoked potential even after the intensive artifact removal procedures. With this effect, the classifier would not have worked normally in an online experiment without a visual cue.

#### 4.6.4. Subjective Assessment

The resultant NASA-TLX showed that most of the participants’ mental demand, temporal demand, effort, and frustration scores increased in the second experiment compared to the first, while performance decreased. The two-tailed Student’s t-test results showed that mental demand and frustration significantly increased, while performance significantly decreased compared to the first experiment. These results also help explain the low classification accuracy because higher mental demand and temporal demand cause mental fatigue, which results in lower performance [94]. To address this issue, [95] set a series of exercises for participants to eat ice cream by MI and an external device, and the authors confirmed that the workload was greatly reduced. These results suggested that the BCI experiment should be more interesting, and, as suggested by the participants in the post-experiment questionnaire, an attempt to reduce the tedium and length of the MI training period may be necessary.

In the post-experiment questionnaire, some participants complained of either shoulder or upper arm muscle fatigue instead of the forearm. There are three possible reasons to explain shoulder muscle fatigue. First, the participants were asked to move their forearm to hold and move a ball during the experiment. In order to perform these sequential tasks, stretching movements of the upper arm and shoulder must be accompanied. These movement may lead to shoulder muscle fatigue because the stretching movement could cause muscle pain due to the spastic muscles [96]. Another reason is that unnatural responses to FES might cause muscle fatigue. Although the current amplitude of FES was gradually increased by utilizing the ramp time function, some patient showed the retraction of the shoulder as an adverse reaction to electrical stimulation. This unnecessary tension of the body may have caused fatigue of the shoulder and upper arm muscles. Finally, this may be due to the awkward posture caused by the wires in the BCI-FES system, which limited the movement of the patients.

## 5. General Discussion

### 5.1. SMR-Based BCI Systems for a 2-Class MI Task in a Single Hand

In this study, relatively new concepts of MI tasks were tested to build a novel SMR-based BCI system that could support a 2-class MI task in a single hand. Most SMR-based BCI research are categorized into two groups. The first group utilized SMR features to initiate a 1-dimensional application similar to an on/off switch [41,97], while the other group controlled a 2-dimensional application such as the left/right, up/down, go/back motions of a robot, a wheelchair, a browser, and a game [98,99,100]. This is because the characteristic of the SMR features induced by MIs is most distinct only near the contralateral motor cortex in the left and right hemispheres.

Due to these limitations, the MI study has made many efforts to increase the classes (beyond left and right). A common example is the use of MI together with visual stimuli, also known as hybrid BCI, for browsers [99] or e-mail clients [100]. In this case, a monitor that can display visual stimuli can be used without restriction because it is a required device of browsers or e-mail clients. However, it is not natural to use visual stimuli to control upper or lower extremities using an exoskeleton, orthosis, or FES. In addition, it is awkward to employ imaginary movements of left and right hands to implement two movements in a single hand. Therefore, it is very important to study the two movements in a single hand as in this study.

However, few studies explored distinct SMR features induced by different rhythmic MIs. [66] investigated four MI tasks consisting of two MI types (wrist extension and rotation) and two speeds (fast and slow), and the authors reported that the speed variable had greater classification ability than the MI types. Similarly, [65] employed slow-continuous and fast-transient MI tasks to classify a 2-class MI task in a single hand such as grasping or opening. Therefore, in this experiment, the different speed MI tasks were utilized to elicit the discriminative SMR patterns. As the MI task is a mental imagination, clear MI instruction is essential for participants to follow different hand movements at the correct speed and timing. To address this issue, video clips were utilized to train the exact motion and speed to participants [101,102]. Although visual cues explain the tasks easily, this method also has a critical issue: a strong visual artifact. Therefore, elaborate removal methods are required. After participants had completed training, their brain signals were analyzed to extract two important features, such as the features to initiate the BCI system and to classify the MI tasks. In this study, the ERD/ERS and CSP methods were used with the sub-band division procedure. The advantages of the sub-band method are (1) the efforts for feature selection can be minimized by automated weighing techniques, and (2) wide frequency bands can be utilized. However, the disadvantage of this method is computational time, because all combinations of frequency bands and channels (or time bins) should be separately classified. In addition, to address the limitations of the SMR-based BCI such that (1) a long training period is required, and (2) the classification accuracy of SMR is relatively lower than visual-based BCIs [83], the ensemble method can be utilized by fusing multiple classifiers, such as LDA and SVM methods, to make one better and/or robust decision similar to this study.

Once the offline analysis is completed, the BCI system and algorithms should be tested under the online condition. Let us assume one moderate classifier that has 70% accuracies for both detecting SMR and classifying ACT will be used. Then, the expected accuracy for a certain target without error would be with a 49% chance (0.7 × 0.7 = 0.49) with a 30% chance in idle status (no SMR was detected) and a 21% chance in wrong status. If any mistake occurs, then the same procedure and chances will follow. For this reason, there is a great need for a new type of training and practical experiment methods that can alleviate this problem—for example, fixed sequence training under the semi-asynchronous mode, which was proposed in this study.

### 5.2. Semi-Asynchronous Mode

**Figure 3:**
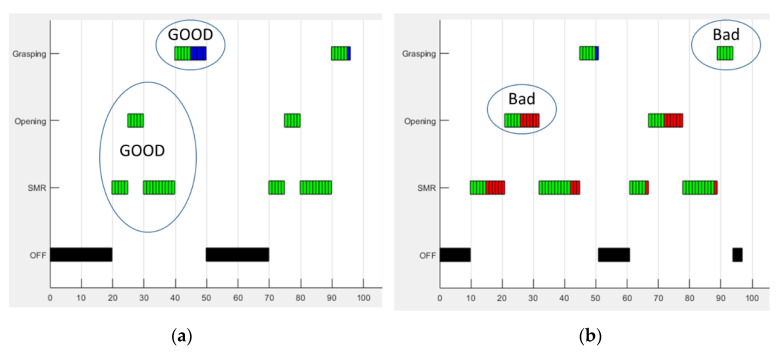
Graphical representations of good (**a**) and bad (**b**) trials.

Figure 3 illustrates the results of good and bad trials. The x-axis of the diagram shows time in seconds, and the y-axis displays the current FES status. Black boxes indicate the waiting time given at the beginning or between sequences. Green boxes indicate the time when the BCI system does not classify in the 5 s given immediately after performing the MI task between periods. Red boxes mean incorrect classification results, and blue boxes imply the time that successfully performed the keep-FES task. This box should appear for 5 s, meaning it was done perfectly without mistakes (5 correct decisions), unlike the red box.

Figure 3a is an example of a successful trial that not only shows a balanced overall accuracy but also does not make any consecutive incorrect decisions. However, as an example of low performance, the right graph shows the successive failure of the opening-MI task, and the FES system automatically performed the opening task in accordance with the 6-s maximum analysis time. In addition, the two green boxes at the top right indicate that the participant did not successfully perform the keep-FES task, and there is only one blue bar, meaning one-time success. Five seconds after the opening-MI task was started, the participants should be in a calm state without any imagination to stop FES. Figure 3b indicates that the stop-FES task was not normally performed, since many red bars appear, as shown in the figure.

However, under the complete asynchronous mode, it is difficult to know which timing shows the onset of MI by users due to the nature of the self-paced experiment. Furthermore, participants also get confused if errors are accumulated and easily get frustrated with low performance, especially for disabled groups. Therefore, a novel experiment design was proposed that tried to achieve the advantages of both modes by addressing the disadvantages of each. As a result, users successfully finished the experiment without excessive delay. Furthermore, the adaptive learning method was also applied under the proposed mode which was not feasible or difficult to apply in asynchronous mode. Thus, in this experiment, fixed sequence training in the semi-asynchronous mode was proposed. This mode has three important features that combined both synchronous and asynchronous methods as follows: (1) the sequence of tasks is fixed to clarify what the user should do; (2) there are error-free results to prevent them from being confused or exhausted, and (3) it utilizes an SMR detecting initiation module similar to the asynchronous mode. Under the proposed semi-asynchronous mode, participants would be free from confusion and frustration. Furthermore, the experimenter could utilize the brain signal under the semi-asynchronous mode to enhance the classification algorithms by adjusting the classification parameters based on the most recent data, which is also known as adaptive machine learning [85,86].

The outcome of this study was the construction of a multi-class SMR-based BCI system with natural MI tasks, as well as restoring the motor functions of disabled individuals. The results of Studies 1 and 2 were to address the low classification accuracy with electrical stimulation by applying the adaptive learning method under semi-asynchronous mode. Then, the proposed experiment mode plays an important role to bridge the gap between the two modes. Furthermore, this approach can not only improve task performance by minimizing the misclassification rate but can also enhance user performance by increasing self-esteem and reducing frustration.

## 6. Conclusion and Future Research

The results of the present study showed that the application of a user-specific FES parameter significantly reduced perceived muscle pain and discomfort compared to a fixed parameter used in the previous studies. Furthermore, all participants were able to classify a 2-class MI task in a single hand by utilizing two different rhythmic MIs, including SOG and FCO, and the ensemble classification method was able to classify a 2-class MI task significantly higher than the LDA method. Finally, the application of the adaptive learning method significantly increased the classification accuracy of the proposed SMR-based BCI-controlled FES system with fixed sequence training under semi-asynchronous mode.

### 6.1. Contributions to BCI-FES Research

A few BCI research studies have investigated a 2-class MI by applying different MI task types such as different speeds and motions, and some research studies used FES systems for stroke and TBI patients. However, this study was one of the few studies to apply both the FES system and the BCI system with a 2-class MI task in a single hand for stroke and TBI patients. Furthermore, in this study, the semi-asynchronous mode with the fixed sequence was proposed that combines the advantages of both synchronous and asynchronous modes. The advantages of the proposed semi-asynchronous mode are (1) participants would be free from confusion and frustration because there is no error; (2) the experimenter can utilize the brain signals under the semi-asynchronous mode to enhance the classification algorithms, and (3) it is easy to change to two modes, both synchronous and asynchronous, with a small modification.

### 6.2. Research Implications in Human Factor and Ergonomics (HF/E)

The outcomes of this study have four important HF/E implications. First, both the proposed FES platform and the SMR-based BCI system can be utilized as a test bed to investigate important HF/E topics such as the effects of individual differences, environmental factors, task performance, and user satisfaction. Second, assistive technologies could be developed and connected to the SMR-based BCIs, such as robot controls, communication, and entertainment games, which would make it possible to explore multitudes of HF/E topics using BCI technology. Third, the SMR-based BCI-controlled FES systems can connect those with motor disabilities (e.g., stroke and TBI patients) to other people, greatly improving their quality of life, enhancing ADL capacity, and even increasing self-esteem. Finally, the results of the study are expected to be utilized to gain an understanding of neuroplasticity in the musculoskeletal rehabilitation of stroke and TBI patients in clinical studies.

### 6.3. Research Limitation and Future Work

The main limitation of this study was that the sample size was too small to have enough statistical power. If additional experiments are conducted, some main effects that we concluded to be not significant are likely to change. The small sample size also prevented the expansion of the study, such as investigating differences in performance according to severity and differences between stroke and TBI patients. With a small sample size, caution must be applied, as the findings might not be transferable to other BCI user groups.

Two types of MI such as kinesthetic motor imagery (KMI) and visual motor imagery (VMI) have been widely used in the BCI research community. Studies have showed that their subjective evaluation for vividness and the amount of shared cortical circuits corresponding with motor execution may each differ across individuals [103]. More research utilizing kinesthetic MI is still required. The classification accuracies during the FES period for both asynchronous and semi-asynchronous experiments were not higher than the true chance level, and it implies that the proposed SMR-based BCI system could not distinguish between voluntary MI-evoked SMRs and FES-driven passive-movement-evoked SMRs. There are three possible reasons to explain this low accuracy. First, when FES was activated, the electrical artifacts might be not completely grounded by the anti-static wrist strap applied to the opposite hand of the affected forearm. Thus, it is possible that electrical stimulation was mixed with the EEG signals, making it difficult to classify. Second, these passive movements driven by FES might have prevented the patients from stopping the MI tasks, as some participants mentioned that it was difficult to stay calm without movement-related imagination when FES was activated. Finally, although the participants were asked not to perform any voluntary physical movements during the experiments, they might simultaneously perform spontaneous movements by following the FES-driven motion. Since EMG signals were not measured in this study, it was not known whether the motion had progressed. Therefore, this limitation should be solved through further study.

Although the classification accuracy of the No-FES period after applying adaptive learning under semi-asynchronous mode was significantly higher than the true chance level, it did not achieve the threshold level (70%) for an effective control through a BCI system [92]. Moreover, the adaptive learning method significantly increased the accuracy similar to other studies [61], but these results might be due to an order effect that the participants were accustomed to the semi-asynchronous experiment while continuing to conduct the experiment. The other limitation was that adequate FES electrode placement was altered during supination and pronation, leading to sharp muscle pain. Participants were asked to minimize supination and pronation to prevent any muscle pain and discomfort; however, this restriction made their movement unnatural. As [104] reviewed, the array types of FES electrode are available, which could help to solve this issue by synchronizing between supination (pronation) and the electrode onset in real time. Finally, this study did not consider the reinforcement or attenuation of SMRs over time. It might be possible to identify the change of SMRs as the time progressed while conducting a long-time experiment, and the relationship between the time and MI task performance could be evaluated to determine the proper duration of the SMR-based BCI experiment with respect to the fatigue and tiredness of participants. In this way, task performance can be maximized, and attention deterioration can be minimized.

## Figures and Tables

**Table 1:** Accuracy of each period and classification methods (%).

	SMR Period			ACT Period			FES Period		
	LDA	SVM	Ensemble	LDA	SVM	Ensemble	LDA	SVM	Ensemble
S01	56.29	56.31	59.17	64.04	65.45	69.51	46.29	50.52	48.03
S02	74.31	73.97	76.03	70.23	67.99	73.94	53.71	63.18	58.71
S03	74.28	74.68	76.44	69.30	68.07	70.26	60.00	66.67	62.50
S04	84.06	81.92	83.62	66.40	67.50	70.15	55.05	54.38	53.55
S05	67.90	64.92	67.50	58.75	63.07	64.24	54.85	60.23	55.83
S06	67.23	65.51	68.04	70.27	73.64	76.59	53.33	62.73	61.74
S07	75.48	78.23	79.11	66.52	70.27	73.91	58.64	59.85	60.91
S08	75.00	74.50	78.00	70.20	70.02	71.43	73.00	72.18	74.00
Mean	71.82	71.26	73.49	66.96	68.25	71.25	56.86	61.22	59.41
SD	7.61	7.81	7.42	3.78	2.99	3.49	7.20	6.33	7.13

FES: Functional Electrical Stimulation, SMR: sensorimotor rhythm. SD: standard deviation

**Table 2:** Average completion rate for each task (%).

	Grasping	Opening	No-FES	Keep	Stop	Yes-FES	Grand
			Average			Average	Average
S01	71.7	70.8	71.3	31.7	68.3	50.0	60.6
S02	71.7	60.8	66.3	26.7	70.0	48.3	57.3
S03	38.3	29.2	33.8	40.0	84.2	62.1	47.9
S04	42.5	93.3	67.9	13.3	95.0	54.2	61.0
S05	78.3	50.0	64.2	90.0	71.7	80.8	72.5
S06	73.3	72.5	72.9	52.5	59.2	55.8	64.4
S07	45.0	55.0	50.0	68.3	22.5	45.4	47.7
S08	65.0	90.0	77.5	51.7	40.0	45.8	61.7
SD	15.0	19.8	13.4	22.9	21.8	11.0	19.9
Average	55.7	65.2	63.0	46.8	63.9	55.3	

**Table 3:** Average completion rate of each independent variable (IV) (%).

	Before Learning			After Learning			
	No-FES	Yes-FES	Average	No-FES	Yes-FES	Average	Grand
S01	71.7	49.2	60.4	70.8	50.0	60.4	60.4
S02	57.5	39.2	48.3	75.0	48.3	61.7	55.0
S03	44.2	62.5	53.3	23.3	62.1	42.7	48.0
S04	55.8	49.2	52.5	80.0	54.2	67.1	59.8
S05	58.3	72.5	65.4	70.0	80.8	75.4	70.4
S06	73.3	50.0	61.7	72.5	55.8	64.2	62.9
S07	40.0	43.3	41.7	60.0	45.4	52.7	47.2
S08	46.7	61.7	54.2	45.0	65.0	55.0	54.6
SD	11.38	10.41	7.19	17.80	10.73	9.26	7.25
Average	55.94	53.44	54.69	62.08	57.71	59.90	

**Table 4:** Accuracy of each task (%).

	Grasping	Opening	No-FES	Keep	Stop	Yes-FES	Grand
			Average			Average	Average
S01	60.0	55.0	57.5	50.0	45.0	47.5	52.5
S02	65.0	55.0	60.0	35.0	55.0	45.0	52.5
S03	30.0	35.0	32.5	50.0	60.0	55.0	43.8
S04	40.0	85.0	62.5	15.0	80.0	47.5	55.0
S05	70.0	45.0	57.5	90.0	45.0	67.5	62.5
S06	70.0	60.0	65.0	60.0	55.0	57.5	61.3
S07	35.0	50.0	42.5	75.0	10.0	42.5	42.5
S08	65.0	70.0	67.5	65.0	10.0	37.5	52.5
SD	15.5	14.3	11.2	21.8	22.6	8.9	6.7
Average	54.4	56.9	55.6	55.0	45.0	50.0	

**Table 5:** Accuracy of each IV (%).

	Before Learning			After Learning			
	No-FES	Yes-FES	Average	No-FES	Yes-FES	Average	Grand
S01	55.0	45.0	50.0	60.0	50.0	55.0	55.0
S02	50.0	30.0	40.0	70.0	60.0	65.0	65.0
S03	30.0	55.0	42.5	35.0	55.0	45.0	45.0
S04	55.0	40.0	47.5	70.0	55.0	62.5	62.5
S05	50.0	55.0	52.5	65.0	80.0	72.5	72.5
S06	60.0	55.0	57.5	70.0	60.0	65.0	65.0
S07	30.0	40.0	35.0	55.0	45.0	50.0	50.0
S08	65.0	40.0	52.5	70.0	35.0	52.5	52.5
SD	12.1	8.7	7.0	11.4	12.2	8.7	8.7
Average	49.4	45.0	47.2	61.9	55.0	58.4	58.4

**Table 6:** Information Transfer Rate (ITR) for each IV (bit/min).

	Before Learning			After Learning			Grand
	No-FES	Yes-FES	Average	No-FES	Yes-FES	Average	
S01	13.12	12.71	12.91	12.87	13.03	12.95	12.93
S02	11.81	14.13	12.97	12.24	13.47	12.86	12.91
S03	10.80	12.39	11.60	14.03	9.53	11.78	11.69
S04	11.68	15.91	13.79	14.96	14.03	14.50	14.14
S05	11.88	10.69	11.29	10.47	12.95	11.71	11.50
S06	13.29	10.10	11.70	13.29	13.20	13.25	12.47
S07	10.52	10.10	10.31	9.40	12.03	10.71	10.51
S08	13.47	10.52	12.00	11.10	14.03	12.56	12.28
Average	12.07	12.07	12.07	12.29	12.78	12.54	12.30

**Table 7:** Results of t-test for each question.

	Estimate	SD	t Ratio	Pr. > |t|
Mental Demand	3.5	1.4516	2.41	0.0467*
Temporal Demand	3.125	1.574773	1.98	0.0876
Performance	−4.25	1.485044	−2.86	0.0243*
Effort	0.25	1.346291	0.19	0.858
Frustration	6.25	1.644797	3.8	0.0067*

* Probability (Pr.) < 0.05.

## References

[1] Van Delden A.E.Q., Peper C.E., Kwakkel G., Beek P.J. (2012). A systematic review of bilateral upper limb training devices for poststroke rehabilitation. Stroke Res. Treat..

[2] Iosa M., Hesse S., Oliviero A., Paolucci S. (2013). New technologies for stroke rehabilitation. Stroke Res. Stroke Res. Treat..

[3] Peckham P.H., Knutson J.S. (2005). Functional electrical stimulation for neuromuscular applications. Annu. Rev. Biomed. Eng..

[4] Sujith O.K. (2008). Functional electrical stimulation in neurological disorders. Eur. J. Neurol..

[5] Johnson L.A., Fuglevand A.J. (2011). Mimicking muscle activity with electrical stimulation. J. Neural Eng..

[6] Popovic M.R., Curt A., Keller T., Dietz V. (2001). Functional electrical stimulation for grasping and walking: Indications and limitations. Spinal Cord.

[7] Wolpaw J.R., Birbaumer N., McFarland D.J., Pfurtscheller G., Vaughan T.M. (2002). Brain-computer interfaces for communication and control. Clin. Neurophysiol..

[8] Daly J.J., Wolpaw J.R. (2008). Brain-computer interfaces in neurological rehabilitation. Lancet Neurol..

[9] Pfurtscheller G., Müller G.R., Pfurtscheller J., Gerner H.J., Rupp R. (2003). “Thought”—Control of functional electrical stimulation to restore hand grasp in a patient with tetraplegia. Neurosci. Lett..

[10] McGie S.C., Zariffa J.J., Popovic M.R., Nagai M.K. (2015). Short-term neuroplastic effects of brain-controlled and muscle-controlled electrical stimulation. Neuromodulation.

[11] Rohm M., Muller-Putz G.R., Kreilinger A., Von Ascheberg A., Rupp R. (2010). A hybrid-Brain Computer Interface for control of a reaching and grasping neuroprosthesis. Biomed. Tech..

[12] Hara Y. (2008). Neurorehabilitation with new functional electrical stimulation for hemiparetic upper extremity in stroke patients. J. Nippon. Med Sch..

[13] Mozaffarian D., Benjamin E.J., Go A.S., Arnett D.K., Blaha M.J., Cushman M., Das S.R., De Ferranti S., Despres J.-P., Fullerton H.J. (2015). Heart Disease and Stroke Statistics—2016 Update: A Report From the American Heart Association. Circulation.

[14] Hirtz D., Thurman D., Gwinn-Hardy K., Mohamed M., Chaudhuri A., Zalutsky R. (2007). How common are the “common” neurologic disorders?. Neurology.

[15] Pereira S., Teasell R., Graham R., Salter K. (2013). Rehabilitation of Severe Stroke. Evid. Based Rev. Stroke Rehabil. Module.

[16] Miller E.L., Murray L., Richards L., Zorowitz R.D., Bakas T., Clark P., Billinger S.A. (2010). Comprehensive overview of nursing and interdisciplinary rehabilitation care of the stroke patient: A scientific statement from the American heart association. Stroke.

[17] Wissel J., Schelosky L.D., Scott J., Christe W., Faiss J., Mueller J. (2010). Early development of spasticity following stroke: A prospective, observational trial. J. Neurol..

[18] Langhorne P., Coupar F., Pollock A. (2009). Motor recovery after stroke: A systematic review. Lancet Neurol..

[19] Duncan P.W., Zorowitz R.D., Bates B., Choi J.Y., Glasberg J.J., Graham G.D., Katz R.C., Lamberty K., Reker D. (2005). Management of Adult Stroke Rehabilitation Care. Stroke.

[20] Matsumoto S., Shimodozono M., Etoh S., Shimozono Y., Tanaka N., Kawahira K. (2010). Beneficial effects of footbaths in controlling spasticity after stroke. Int. J. Biometeorol..

[21] Kawashima N., Popovic M.R., Zivanovic V. (2013). Effect of Intensive Functional Electrical Stimulation Therapy on Upper-Limb Motor Recovery after Stroke: Case Study of a Patient with Chronic Stroke. Physiother. Can..

[22] Quandt F., Hummel F.C. (2014). The influence of functional electrical stimulation on hand motor recovery in stroke patients: A review. Exp. Transl. Stroke Med..

[23] Lynch C.L., Popovic M.R. (2008). Functional electrical stimulation. IEEE Control Syst..

[24] Levin M.F., Hui-Chan C.W. (1992). Relief of hemiparetic spasticity by TENS is associated with improvement in reflex and voluntary motor functions. Electroencephalogr. Clin. Neurophysiol. Potentials Sect..

[25] Sabut S.K., Sikdar C., Kumar R., Mahadevappa M. (2011). Functional electrical stimulation of dorsiflexor muscle: Effects on dorsiflexor strength, plantarflexor spasticity, and motor recovery in stroke patients. Neurorehabilitation.

[26] Zhang D., Guan T.H., Widjaja F., Ang W.T. (2007). Functional electrical stimulation in rehabilitation engineering: A Survey. i-CREATe ’07: Proceedings of the 1st International Convention on Rehabilitation Engineering & Assistive Technology in Conjunction with 1st Tan Tock Seng Hospital Neurorehabilitation Meeting.

[27] Kottink A.I.R., Oostendorp L.J., Buurke J.H., Nene A.V., Hermens H.J., Ijzerman M.J. (2004). The Orthotic Effect of Functional Electrical Stimulation on the Improvement of Walking in Stroke Patients with a Dropped Foot: A Systematic Review. Artif. Organs.

[28] Jackson A., Mavoori J., Fetz E.E. (2006). Long-term motor cortex plasticity induced by an electronic neural implant. Nature.

[29] Teasell R., Bayona N.A., Bitensky J. (2005). Plasticity and Reorganization of the Brain Post Stroke. Top. Stroke Rehabil..

[30] Papachristos A. (2014). Functional Electrical Stimulation in Paraplegia. Topics in Paraplegia.

[31] Young B.M., Nigogosyan Z., Nair V.A., Walton L.M., Song J., Tyler M.E., Edwards D.F., Caldera K., Sattin J.A., Williams J.C. (2014). Case report: Post-stroke interventional BCI rehabilitation in an individual with preexisting sensorineural disability. Front. Neuroeng..

[32] Chae J., Sheffler L.R., Knutson J.S. (2008). Neuromuscular Electrical Stimulation for Motor Restoration in Hemiplegia. Top. Stroke Rehabil..

[33] Lawrence M. (2009). Transcutaneous Electrode Technology for Neuroprostheses. Ph.D. Thesis.

[34] Mangold S., Keller T., Curt A., Dietz V. (2004). Transcutaneous functional electrical stimulation for grasping in subjects with cervical spinal cord injury. Spinal Cord.

[35] Draganski B., Gaser C., Busch V., Schuierer G., Bogdahn U., May A. (2004). Neuroplasticity: Changes in grey matter induced by training. Nature.

[36] Lourenção M.I.P., Battistella L.R., De Brito C.M.M., Tsukimoto G.R., Miyazaki M.H. (2008). Effect of biofeedback accompanying occupational therapy and functional electrical stimulation in hemiplegic patients. Int. J. Rehabil. Res..

[37] Brouwer A.-M., Van Erp J.B.F. (2010). A tactile P300 brain-computer interface. Front. Mol. Neurosci..

[38] Mak J.N., Wolpaw J.R. (2009). Clinical Applications of Brain-Computer Interfaces: Current State and Future Prospects. IEEE Rev. Biomed. Eng..

[39] Käthner I., Ruf C.A., Pasqualotto E., Braun C., Birbaumer N., Halder S. (2013). A portable auditory P300 brain–computer interface with directional cues. Clin. Neurophysiol..

[40] Barsi G.I., Popović D.B., Tarkka I.M., Sinkjær T., Grey M.J. (2008). Cortical excitability changes following grasping exercise augmented with electrical stimulation. Exp. Brain Res..

[41] Pfurtscheller G., Solis-Escalante T., Ortner R., Linortner P., Müller-Putz G.R. (2010). Self-Paced Operation of an SSVEP-Based Orthosis with and Without an Imagery-Based “Brain Switch”: A Feasibility Study Towards a Hybrid BCI. IEEE Trans. Neural Syst. Rehabil. Eng..

[42] Holz E.M., Botrel L., Kübler A. (2015). Independent home use of Brain Painting improves quality of life of two artists in the locked-in state diagnosed with amyotrophic lateral sclerosis. Brain Comput. Interfaces.

[43] Li Y., Nam C.S. (2015). A Collaborative Brain-Computer Interface (BCI) for ALS Patients. Proc. Hum. Factors Ergon. Soc. Annu. Meet..

[44] Nam C.S., Lee J., Bahn S., Li Y., Choi I. Brain-Computer Interface Supported Collaborative Work. Proceedings of the 5th International Brain-Computer Interface Meeting.

[45] Nam C.S., Moore M., Choi I., Li Y. (2015). Designing Better, Cost-Effective Brain–Computer Interfaces. Ergon. Des. Q. Hum. Factors Appl..

[46] Bhattacharyya S., Khasnobish A., Ghosh P., Mazumder A., Tibarewala D.N. (2015). A Review on Brain Imaging Techniques for BCI Applications. Medical Imaging: Concepts, Methodologies, Tools and Applications.

[47] Homer M.L., Nurmikko A.V., Donoghue J.P., Hochberg L.R. (2013). Implants and Decoding for Intracortical Brain Computer Interfaces. Annu. Rev. Biomed. Eng..

[48] Amiri S., Rabbi A., Azinfar L., Fazel-Rezai R., Asadpour V. (2013). A Review of P300, SSVEP, and Hybrid P300 / SSVEP Brain- Computer Interface Systems. Brain Comput. Interfaces Syst..

[49] Beisteiner R., Höllinger P., Lindinger G., Lang W., Berthoz A. (1995). Mental representations of movements. Brain potentials associated with imagination of hand movements. Electroencephalogr. Clin. Neurophysiol. Potentials Sect..

[50] Müller G.R., Neuper C., Rupp R., Keinrath C., Gerner H.J., Pfurtscheller G. (2003). Event-related beta EEG changes during wrist movements induced by functional electrical stimulation of forearm muscles in man. Neurosci. Lett..

[51] Neuper C., Wörtz M., Pfurtscheller G. (2006). ERD/ERS patterns reflecting sensorimotor activation and deactivation. Prog. Brain Res..

[52] Pfurtscheller G. (1977). Graphical display and statistical evaluation of event-related desynchronization (ERD). Electroencephalogr. Clin. Neurophysiol..

[53] Pfurtscheller G. (1992). Event-related synchronization (ERS): An electrophysiological correlate of cortical areas at rest. Electroencephalogr. Clin. Neurophysiol..

[54] Zhang Y., Nam C.S., Zhou G., Jin J., Wang X., Cichocki A. (2018). Temporally constrained sparse group spatial patterns for motor imagery BCI. IEEE trans. cyber..

[55] Daly J.J., Cheng R., Rogers J., Litinas K., Hrovat K., Dohring M. (2009). Feasibility of a New Application of Noninvasive Brain Computer Interface (BCI): A Case Study of Training for Recovery of Volitional Motor Control After Stroke. J. Neurol. Phys. Ther..

[56] Takahashi M., Takeda K., Otaka Y., Osu R., Hanakawa T., Gouko M., Ito K. (2012). Event related desynchronization-modulated functional electrical stimulation system for stroke rehabilitation: A feasibility study. J. Neuroeng. Rehabil..

[57] Sharma N., Pomeroy V.M., Baron J.C. (2006). Motor imagery: A backdoor to the motor system after stroke?. Stroke.

[58] De Vries S., Mulder T. (2007). Motor imagery and stroke rehabilitation: A critical discussion. J. Rehabil. Med..

[59] Krusienski D.J., Grosse-Wentrup M., Galán F., Coyle D., Miller K.J., Forney E., Anderson C.W. (2011). Critical issues in state-of-the-art brain–computer interface signal processing. J. Neural Eng..

[60] Lotte F., Congedo M., Lecuyer A., Lamarche F., Arnaldi B. (2007). A review of classification algorithms for EEG-based brain–computer interfaces. J. Neural Eng..

[61] Vidaurre C., Blankertz B. (2009). Towards a Cure for BCI Illiteracy. Brain Topogr..

[62] Guger C., Edlinger G., Harkam W., Niedermayer I., Pfurtscheller G. (2003). How many people are able to operate an eeg-based brain-computer interface (BCI)?. IEEE Trans. Neural Syst. Rehabil. Eng..

[63] Gatti R., Tettamanti A., Gough P.M., Riboldi E., Marinoni L., Buccino G. (2013). Action observation versus motor imagery in learning a complex motor task: A short review of literature and a kinematics study. Neurosci. Lett..

[64] Schuster C., Hilfiker R., Amft O., Scheidhauer A., Andrews B., Butler J.A., Kischka U., Ettlin T. (2011). Best practice for motor imagery: A systematic literature review on motor imagery training elements in five different disciplines. BMC Med..

[65] Choi I., Bond K., Krusienski D., Nam C.S. Effects of Off-Site Attention on SSSEP Amplitude. Proceedings of the 6th International Brain-Computer Interface Meeting.

[66] Gu Y., Dremstrup K., Farina D. (2009). Single-trial discrimination of type and speed of wrist movements from EEG recordings. Clin. Neurophysiol..

[67] Ahlstrom V., Kudrick B. (2007). Human Factors Criteria for Displays: A human Factors Design Standard—Update of Chapter 5.

[68] Popović D.B. (2014). Advances in functional electrical stimulation (FES). J. Electromyo. Kinesi..

[69] Schalk G., McFarland D., Hinterberger T., Birbaumer N., Wolpaw J.R. (2004). BCI2000: A General-Purpose Brain-Computer Interface (BCI) System. IEEE Trans. Biomed. Eng..

[70] Delorme A., Makeig S. (2004). EEGLAB: An open source toolbox for analysis of single-trial EEG dynamics including independent component analysis. J. Neurosci. Methods.

[71] Nolan H., Whelan R., Reilly R.B. (2010). FASTER: Fully Automated Statistical Thresholding for EEG artifact Rejection. J. Neurosci. Methods.

[72] Schalk G., Mellinger J. (2010). A Practical Guide to Brain—Computer Interfacing with BCI2000: General-Purpose Software for Brain-Computer Interface Research, Data Acquisition, Stimulus Presentation, and Brain Monitoring.

[73] Pfurtscheller G., Neuper C. (2006). Future prospects of ERD / ERS in the context of brain-computer interface (BCI) developments. Prog. Brain Res..

[74] Golub M.D., Chase S.M., Batista A.P., Yu B.M. (2016). Brain–computer interfaces for dissecting cognitive processes underlying sensorimotor control. Curr. Opin. Neurobiol..

[75] Nam C.S., Nijholt A., Lotte F. (2018). Brain–Computer Interfaces Handbook: Technological and Theoretical Advances.

[76] Pfurtscheller G., Lopes F.H. (1999). Event-related EEG/MEG synchronization and desynchronization: Basic principles. Clin. Neurophysiol..

[77] Novi Q., Guan C., Dat T.H., Xue P. (2007). Sub-band Common Spatial Pattern (SBCSP) for Brain-Computer Interface. Proceedings of the 3rd International IEEE/EMBS Conference on Neural Engineering.

[78] Bashashati A., Fatourechi M., Ward R.K., Birch G.E.E. (2007). A survey of signal processing algorithms in brain–computer interfaces based on electrical brain signals. J. Neural Eng..

[79] Polikar R. (2006). Ensemble based systems in decision making. IEEE Circuits Syst. Mag..

[80] Kohavi R. (1995). A study of cross-validation and bootstrap for accuracy estimation and model selection. IJCAI.

[81] Pfurtscheller G., Neuper C. (2001). Motor imagery and direct brain-computer communication. Proc. IEEE.

[82] Tonet O., Tecchio F., Sepulveda F., Citi L., Rossini P.M., Marinelli M., Tombini M., Laschi C., Dario P. (2006). Critical Review and Future Perspectives of Non-Invasive Brain-Machine Interfaces. https://www.esa.int/gsp/ACT/doc/ARI/ARI%20Study%20Report/ACT-RPT-BIO-ARI-056402-Non_invasive_brain-machine_interfaces_-_Pisa_S%27Anna.pdf.

[83] Ortner R., Allison B.Z., Korisek G., Gaggl H., Pfurtscheller G. (2010). An SSVEP BCI to Control a Hand Orthosis for Persons with Tetraplegia. IEEE Trans. Neural Syst. Rehabil. Eng..

[84] Nicolas-Alonso L.F., Gomez-Gil J. (2012). Brain Computer Interfaces, a Review. Sensors.

[85] Vidaurre C., Sannelli C., Müller K.-R., Blankertz B. (2011). Machine-Learning-Based Coadaptive Calibration for Brain-Computer Interfaces. Neural Comput..

[86] Wolpaw J.R., McFarland D.J. (2004). Control of a two-dimensional movement signal by a noninvasive brain-computer interface in humans. Proc. Natl. Acad. Sci. USA.

[87] Jung T.P., Makeig S., Humphries C., Lee T.W., McKeown M.J., Iragui V., Sejnowski T.J. (2000). Removing electroencephalographic artifacts by blind source separation. Psychophysiology.

[88] Kuiken T.A., Li G., Lock B.A., Lipschutz R.D., Miller L.A., Stubblefield K.A., Englehart K.B. (2009). Targeted Muscle Reinnervation for Real-time Myoelectric Control of Multifunction Artificial Arms. JAMA.

[89] Wodlinger B., Downey J.E., Tyler E.C., Schwartz A.B., Boninger M.L., Collinger J.L. (2014). Ten-dimensional anthropomorphic arm control in a human brain−machine interface: Difficulties, solutions, and limitations. J. Neural Eng..

[90] Wolpaw J.R., Ramoser H., McFarland D., Pfurtscheller G. (1998). EEG-based communication: Improved accuracy by response verification. IEEE Trans. Rehabil. Eng..

[91] Hart S.G., Staveland L.E. (1988). Development of NASA-TLX (Task Load Index): Results of empirical and theoretical research. Adv. Psychol..

[92] Perelmouter J., Birbaumer N. (2000). A binary spelling interface with random errors. IEEE Trans. Rehabil. Eng..

[93] Roset S.A., Gant K., Prasad A., Sanchez J.C. (2014). An adaptive brain actuated system for augmenting rehabilitation. Front. Neurosci..

[94] Fazel-Rezai R., Allison B.Z., Guger C., Sellers E.W., Kleih S.C., Kübler A. (2012). P300 brain computer interface: Current challenges and emerging trends. Front. Neuroeng..

[95] Rohm M., Schneiders M., Müller C., Kreilinger A., Kaiser V., Müller-Putz G.R., Rupp R.R. (2013). Hybrid brain–computer interfaces and hybrid neuroprostheses for restoration of upper limb functions in individuals with high-level spinal cord injury. Artif. Intell. Med..

[96] Vuagnat H., Chantraine A. (2003). Shoulder pain in hemiplegia revisited: Contribution of functional electrical stimulation and other therapies. J. Rehabil. Med..

[97] Blokland Y., Spyrou L., Thijssen D., Eijsvogels T.M., Colier W., Floor-Westerdijk M., Vlek R., Bruhn J., Farquhar J. (2013). Combined EEG-fNIRS Decoding of Motor Attempt and Imagery for Brain Switch Control: An Offline Study in Patients With Tetraplegia. IEEE Trans. Neural Syst. Rehabil. Eng..

[98] Huang D., Qian K., Fei D.-Y., Jia W., Chen X., Bai O. (2012). Electroencephalography (EEG)-Based Brain–Computer Interface (BCI): A 2-D Virtual Wheelchair Control Based on Event-Related Desynchronization/Synchronization and State Control. IEEE Trans. Neural Syst. Rehabil. Eng..

[99] Yu T., Li Y., Long J., Gu Z. (2012). Surfing the internet with a BCI mouse. J. Neural Eng..

[100] Yu T., Li Y., Long J., Li F. (2013). A Hybrid Brain-Computer Interface-Based Mail Client. Comput. Math. Methods Med..

[101] Kim T., Kim S., Lee B. (2015). Effects of Action Observational Training Plus Brain-Computer Interface-Based Functional Electrical Stimulation on Paretic Arm Motor Recovery in Patient with Stroke: A Randomized Controlled Trial. Occup. Ther. Int..

[102] Looned R., Webb J., Xiao Z.G., Menon C. (2014). Assisting drinking with an affordable BCI-controlled wearable robot and electrical stimulation: A preliminary investigation. J. Neuroeng. Rehabil..

[103] Toriyama H., Ushiba J., Ushiyama J. (2018). Subjective Vividness of Kinesthetic Motor Imagery Is Associated with the Similarity in Magnitude of Sensorimotor Event-Related Desynchronization Between Motor Execution and Motor Imagery. Front. Hum. Neurosci..

[104] Keller T., Kuhn A. (2008). Electrodes for transcutaneous (surface) electrical stimulation. J. Autom. Control..

